# Retraction: MicroRNA-613 inhibits cell growth, migration and invasion of papillary thyroid carcinoma by regulating SphK2

**DOI:** 10.18632/oncotarget.28818

**Published:** 2025-12-31

**Authors:** Wangwang Qiu, Zhili Yang, Youben Fan, Qi Zheng

**Affiliations:** ^1^Department of General Surgery, Shanghai Jiao Tong University Affiliated Sixth People’s Hospital, Shanghai 200233, P.R. China; ^*^These authors contributed equally to this work

**This article has been retracted:** Oncotarget has concluded its investigation of the paper following feedback from our readers. We found multiple overlaps and duplications in the published figures, detailed below:

Figure 2C K1 cells transfected with miR-C (K1/miR-C) migration image overlaps with Figure 5C K1/miR-C cells migration, representing different experiment.

Figure 2D TPC-1/miR-C cells migration image overlaps with Figure 5D TPC-1/miR-C cells migration image, representing different experiment.

Figure 5C K1/miR-C cells invasion image overlaps with Figure 5C/miR-C cells migration image.

Figure 5C K1/miR-613 + pcDNA3.1/SphK2 migration image overlaps with Figure 5C K1/miR-613 + pcDNA3.1/SphK2 cells invasion image.

Figure 5D TPC-1/miR-C migration image overlaps with Figure 5D TPC-1/miR-C cells invasion image.

Figure 5D TPC-1/miR-613 invasion image overlaps with Figure 5D TPC-1/miR-613+ pcDNA3.1/SphK2 cells migration image, later corrected.

Figure 5C K1/miR-C cells invasion image overlaps with BCPAP cells transfected with anti-miR-613 invasion image in Supplementary Figure1C.

Figure 5D migration and invasion images (control and miR-613-transfected TPC-1 cells) were reproduced in an unrelated 2018 paper [1] Figure 3C, referring to a different experiment in a different cell line.

Additionally, 6 and 5 tumor images from Figure 3C later appeared in Figure 2E of [2] and Figure 6B of [3], respectively. Both [2] and [3] have since been retracted.

Based on the findings of this investigation, the Oncotarget Editors have decided to retract the article. The authors have been notified of this decision.

Original article: Oncotarget. 2016; 7:39907–39915. 39907-39915. https://doi.org/10.18632/oncotarget.9530
